# Managing High-Risk Liver Transplantation: A Case of Budd-Chiari Syndrome With Preemptive Venovenous Extracorporeal Membrane Oxygenation (ECMO)

**DOI:** 10.7759/cureus.112835

**Published:** 2026-07-17

**Authors:** Mariana Pascoal, Luciane Pereira, Dulce Diogo, Ana Eufrásio

**Affiliations:** 1 Anesthesiology, Unidade Local de Saúde de Coimbra, Coimbra, PRT; 2 Hepatic Transplantation, Unidade Local de Saúde de Coimbra, Coimbra, PRT

**Keywords:** budd-chiari syndrome, extracorporeal membrane oxygenation, hepatic transplantation, hepatic vein thrombosis, liver failure, perioperative care, venovenous extracorporeal membrane oxygenation

## Abstract

We report the use of preemptive dual-cannula venovenous extracorporeal membrane oxygenation (VV-ECMO) during orthotopic liver transplantation in a 21-year-old patient with Budd-Chiari syndrome (BCS) with diffuse hepatic vein thrombosis and inferior vena cava (IVC) narrowing. Progressive liver dysfunction and unsuccessful transjugular intervention led to urgent transplantation. Given the anticipated intraoperative cardiopulmonary instability and surgical complexity, VV-ECMO was initiated before IVC clamping. Classic hepatectomy with portocaval shunt was performed. Portal flow was restored without post-reperfusion syndrome. Protamine was not administered, and no ECMO-related complications occurred. This case highlights the role of preemptive ECMO as a safe strategy in high-risk liver transplantation because of the anticipated severe hemodynamic instability during hepatectomy and IVC clamping due to BCS with extensive hepatic vein thrombosis and IVC stenosis, providing continuous cardiopulmonary support.

## Introduction

Extracorporeal membrane oxygenation (ECMO) has become an important adjunct in high-risk liver transplantation [1]. Historically, ECMO was predominantly employed in life-threatening situations as a salvage method, particularly in potentially reversible respiratory failure and hepatopulmonary syndrome or postoperative support [1-4]. Severe hemodynamic instability, often resulting from post-reperfusion syndrome, are commonly cited reasons for intraoperative ECMO cannulation [4,5].

As clinical experience with ECMO continues to expand, transplant programs are more frequently supporting patients throughout the perioperative period [6]. Extending ECMO support preoperatively may allow liver transplant (LT) for a select group of individuals previously considered ineligible due to cardiopulmonary risk [7]. ECMO now functions as a perioperative bridge to transplantation, and the literature supports its preemptive use for high-risk pulmonary hypertension in patients undergoing LT [1]. Recently, there has been an increase in reports describing ECMO employed as a planned, preventive cardiopulmonary support method to facilitate the procedure [5], as well as intraoperatively for patients experiencing profound hemodynamic compromise [4,7,8]. We describe the preemptive use of venovenous ECMO (VV-ECMO) during orthotopic liver transplantation in a patient with Budd-Chiari syndrome (BCS) to maintain cardiopulmonary stability during inferior vena cava (IVC) clamping. Preemptive VV-ECMO was chosen because of the anticipated severe hemodynamic instability during hepatectomy and IVC clamping due to BCS, with extensive hepatic vein thrombosis and IVC stenosis. It provided continuous cardiopulmonary support and was considered the safest strategy for this high-risk procedure.

BCS is a rare but severe disorder characterized by hepatic venous outflow obstruction, resulting in hepatic congestion, portal hypertension, and progressive hepatic failure if untreated [5,9]. Although anticoagulation and endovascular recanalization may stabilize early disease, orthotopic liver transplantation remains the only curative option for advanced or refractory cases [6,8-10]. Despite its therapeutic potential, LT in BCS is technically challenging due to extensive venous thrombosis, distorted anatomy, and perioperative hemodynamic instability [6,10]. These factors contribute to increased intraoperative blood loss, difficult venous return, and potential right-sided cardiac dysfunction.

## Case presentation

A 21-year-old patient presented with a 1-week history of left flank pain associated with nausea, vomiting, abdominal distension, and constipation. The patient had a history of nonclassical congenital adrenal hyperplasia due to 21-hydroxylase deficiency (mutation in the CYP21A2 gene) and asthma. Her medication was an oral contraceptive.

**Figure 1:**
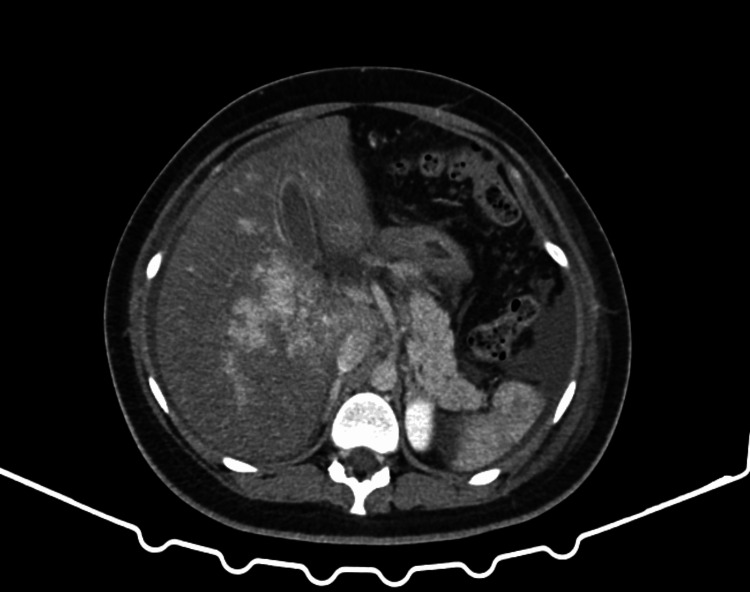
CT scan Hepatomegaly with hypertrophy of segment I and diffuse thrombosis of hepatic veins

The initial investigation demonstrated elevated liver parameters, suggesting hepatocellular injury and cholestasis (Table 1). An ultrasound showed hepatomegaly, moderate ascites, absent flow in the hepatic veins, and hepatopetal flow in the portal vein with reduced velocity, supporting the hypothesis of BCS. The abdominal CT scan (Figure 1) showed hepatomegaly with hypertrophy of segment I, diffuse thrombosis of hepatic veins, patent portal vein with reduced caliber, narrowing of the intrahepatic IVC, and ascites. Anticoagulation was initiated with enoxaparin, and the patient was admitted to a level II care unit. On the third day of hospitalization, the patient developed worsening ascites refractory to medical therapy. An attempt to perform a transjugular intrahepatic portosystemic shunt was unsuccessful. Due to worsening liver function and progressive encephalopathy, scoring a model for end-stage liver disease (MELD)-Na score of 21 [11], the patient was listed for urgent liver transplant.

**Table 1:** Laboratory findings at admission, pretransplant, and at discharge ALT: Alanine Aminotransferase; AP: Alkaline Phosphatase; APTT: Activated Partial Thromboplastin Time; AST: Aspartate Aminotransferase; BUN: Blood Urea Nitrogen; GGT: Gamma-Glutamyl Transferase; INR: International Normalized Ratio; LDH: Lactate Dehydrogenase; PT: Prothrombin Activity; TP: Prothrombin Time

Laboratory parameter	At admission	Pretransplant	At discharge	Reference value
Creatinine	0.61	1.65	0.51	0.61 mg/dL
BUN	8.5	39.0	7.7	7.9 - 20.9 mg/dL
Sodium	133	135	144	136 - 146 mmol/L
Potassium	4.9	5.5	4.9	3.5 - 5.1 mmol/L
Calcium	8.9	9.5	9.7	8.8 - 10.6 mg/dL
Albumin	3.3	2.9	4.0	3.5 - 5.2 g/dL
LDH	438	335	185	< 247 U/L
AST	206	54	26	< 31 U/L
ALT	144	22	42	< 34 U/L
FA	169	95	255	30 - 120 U/L
GGT	37	62	36.5	< 38 U/L
Bilirubin	0.9	1.2	0.5	0.2 - 1.2 mg/dL
TP	14.5	24.9	11.8	9.4 - 12.5 s
PT	73	35	101	70 - 120%
INR	1.21	2.06	0.49	—
APTT	28.6	33.0	24.1	23.4 - 35.4 s
Platelets	421	321	223	150 - 400 x10^9^/L

On arrival in the operating room, a left radial arterial catheter was placed for continuous blood pressure monitoring. General anesthesia was induced, and standard American Society of Anesthesiologists (ASA) monitoring was employed [12], supplemented by bispectral index monitoring [13], train-of-four neuromuscular assessment, urine output measurement, PiCCO (Pulse index Continuous Cardiac Output) arterial pulse-contour analysis, and cerebral oximetry. A left internal jugular central venous catheter was placed under ultrasound guidance.

Venous drainage cannulas consisted of a 21-Fr Getinge cannula (Getinge AB, Göteborg, Sweden) placed in the right femoral vein and a 16-Fr Medtronic cannula (Medtronic plc, Minneapolis, MN, US) inserted into the portal vein. The right internal jugular vein was cannulated with a 17-Fr Getinge cannula for venous return. Systemic anticoagulation was achieved with 5,000 units of unfractioned heparin, with titration guided by viscoelastic testing. Six units of packed red blood cells and 2 gr of fibrinogen were administered intraoperatively.

The graft was obtained from a 46-year-old brain-dead ABO-compatible donor with a body mass index of 27.2 kg/m2. Evaluations were unremarkable. Histological assessment showed macrovesicular steatosis without fibrosis. The Eurotransplant Donor Risk Index was 1.47 [14], and the graft-to-recipient weight ratio was 1.99. The graft underwent 180 min of end-ischemic, hypothermic, oxygenated perfusion with monitoring of PaO2 and perfusate lactate levels (peak lactate 4.19 mmol/L). Hepatectomy was performed using the classic technique, revealing complete fibrotic occlusion of the hepatic veins. A temporary portocaval shunt was created, and VV-ECMO was initiated before IVC clamping. ECMO flows were maintained at 2 L/min.

Implantation included end-to-end reconstruction of the hepatic veins. Recipient portal vein plasty was required. Portal flow was restored without postreperfusion syndrome, allowing discontinuation of ECMO (total duration of 270 min) without the administration of protamine with the support of viscoelastic testing. Arterial and biliary reconstructions were performed as end-to-end anastomoses without placement of a Kehr drain. Graft preservation time was 429 min, with cold and warm ischemia times of 240 min and 69 min, respectively. Total operative time was 455 min. The patient was transferred to the ICU and, on postoperative day one, extubated. No ECMO-related complications or serious adverse events were reported. Discharge was on postoperative day 15, and follow-up indicated biliary stenosis, with good graft function (Table 1).

## Discussion

The use of ECMO in the perioperative management of liver transplantation has evolved from a rescue modality to a selectively planned supportive strategy [5,7,8,10]. Published cases predominantly involved venoarterial ECMO (VA-ECMO) in cardiac dysfunction or pulmonary hypertension [1,7,9], whereas VV-ECMO reports are of respiratory failure or hepatopulmonary syndrome [3,15].

Timing of ECMO initiation is a critical determinant of outcomes in liver transplantation since early ECMO initiation may improve survival and physiological stability, and facilitate successful weaning as compared with salvage deployment [4,7,8]. This case expands the limited experience with preemptive VV-ECMO in liver transplantation for BCS. Although preemptive VV-ECMO has rarely been reported in liver transplantation for BCS, our case demonstrates that it can be a safe and effective strategy to maintain intraoperative hemodynamic stability in selected high-risk patients.

Outcomes of ECMO-supported liver transplantation vary according to indication and timing. Survival is the best in patients supported for isolated respiratory failure and worse in cardiac failure or multiorgan dysfunction, showing no difference in postoperative mortality between VA-ECMO and VV-ECMO [2,6,9,10,15-18].

ECMO may expand transplant eligibility to patients previously considered unsuitable candidates, raising concerns regarding resource utilization and organ allocation [1,7,8,10]. Careful patient selection is critical. Favorable candidates have reversible respiratory failure, preserved cardiac function, without uncontrolled infection, and acceptable neurological status [4,7,8,10]. Patients with BCS are particularly vulnerable to intraoperative instability, making preemptive VV-ECMO an attractive strategy [18].

Also, ECMO may facilitate transplantation by decompressing the IVC and portal circulation, avoiding prolonged IVC clamping and promoting hemodynamic stability. Potential benefits include reducing ischemia-reperfusion injury, blood loss, respiratory complications, and incidence of acute kidney injury in the case of preexisting renal dysfunction, therefore enhancing recipient and graft outcomes [6].

The main disadvantages of ECMO in liver transplantation include longer operative times, requiring expertise, specialized equipment, and ECMO-related complications in LT recipients, which include bleeding, thrombosis, infection, hemolysis, and vascular injury [5,7,16,18]. Bleeding remains the most common complication, particularly in cirrhotic patients with baseline coagulopathy and perioperative anticoagulation requirements [7,8]. VV-ECMO is associated with lower rates of limb ischemia and embolic events compared with VA-ECMO, although hemorrhagic complications remain significant [5,10]. 

As a strategy, cannulation should be as far as possible from the vascular anastomoses, ensuring adequate venous drainage while minimizing excessive negative pressures, which increase vessel injury, air entrainment, and bleeding [6,7]. High-volume, low-pressure inflow and outflow cannulas are recommended [6].

For VV-ECMO, dual-lumen cannulas are discouraged. Since the right internal jugular vein is favored for cannulation, multidisciplinary planning is recommended [7]. Typically, a femoral venous cannula is placed for drainage, with blood returned via a cannula in the internal jugular vein. When portal bypass is indicated, an additional portal vein drainage cannula is inserted [6]. Early decannulation is associated with improved outcomes and reduced ECMO-related morbidity [4,16,17]. Decannulation is considered with adequate gas exchange, hemodynamic stability, and acceptable graft function [7,8].

Anticoagulation during ECMO support in liver transplantation is challenging because of the competing risks of bleeding and thrombosis in cirrhosis and ECMO. No consensus strategy exists for LT recipients [19]. Although systemic anticoagulation is required to prevent circuit thrombosis, it must be carefully balanced against the high risk of perioperative bleeding, especially in the early postoperative period. Viscoelastic testing is typically used to guide its management, with intravenous heparin favored due to its rapid onset and reversibility (50-100 U/kg during cannulation). Anticoagulation targets and heparin reversal with protamine should be individualized, guided by viscoelastic tests and surgical field assessment. Excessive protamine administration may precipitate hypercoagulability and thrombosis, particularly in the context of hemorrhage, ongoing resuscitation, and deficiencies of endogenous anticoagulants such as protein C and S. Hemostatic balance may fluctuate after graft reperfusion due to transient release of endogenous heparinoids; increased bleeding risk despite its excessive correction may predispose to thrombosis [7].

Several limitations should be acknowledged. This is a single case from one center, and the individual contributions of VV-ECMO and surgical technique to the observed outcomes cannot be disentangled. Publication bias toward successful cases must also be considered. Future prospective registry studies with comparable patient populations and long-term follow-up are required to establish evidence-based protocols, cannulation, and anticoagulation strategies for ECMO-supported LT.

## Conclusions

The role of ECMO in liver transplantation continues to evolve, with increasing interest in its preemptive role in highly selected patients. Advances in cannulation techniques, anticoagulation management, and multidisciplinary perioperative planning are likely to expand the safe use of ECMO in transplantation to facilitate the surgical technique and improve patient and graft outcomes. In BCS, where complex hemodynamic and respiratory interactions are common, preemptive VV-ECMO may represent a valuable adjunct in selected cases. This case contributes to the limited but growing body of evidence supporting proactive ECMO strategies and underscores the need for prospective large-scale studies to define optimal selection criteria and protocols related to cannulation strategies and anticoagulation management.
